# Laparoscopic Roux-en-Y Gastric Bypass: Evolution of Weight Loss and Metabolic Obesity-Related Complications After 15–20 Years

**DOI:** 10.1007/s11695-025-08037-7

**Published:** 2025-07-09

**Authors:** Beatriz Barberá-Carbonell, Anna Dayer-Jankechova, Sergio Gaspar-Figueiredo, Heloise Smet, Styliani Mantziari, Michel Suter

**Affiliations:** 1 Riviera-Chablais Hospital, Rennaz, Switzerland; 2 Present Address: University Hospital (CHUV), Lausanne, Switzerland; 3 Faculty of Biology and Medicine, University of Lausanne, Lausanne, Switzerland

**Keywords:** Obesity, Gastric bypass, Roux-en-Y gastric bypass, Bariatric surgery, Metabolic surgery, Long-term

## Abstract

**Background:**

Roux-en-Y gastric bypass (RYGB) is one of the prevailing metabolic/bariatric surgical (MBS) procedures. It has been used for > 50 years, yet few long-term results (> 10 years) have been published. The aim of this study is to report 15-year results and beyond in a large patient group regarding weight loss, metabolic outcomes and long-term morbidity.

**Methods:**

Our prospective bariatric database was reviewed and analyzed retrospectively. All patients who underwent primary laparoscopic RYGB between 1999 and 2008 were included. Data was gathered during in-person visits for most patients, but also using electronic medical records, phone calls to patients and/or general practitioners.

**Results:**

Nine hundred forty-four patients underwent RYGB during the study period. All were eligible for follow-up after 15 years, and 340 were eligible after 20 years. 39 (4.1%) patients died during the first 15 postoperative years. Follow-up rates were 91.7%, 74.5% and 52.3% after 10, 15 and 20 years respectively. Weight loss peaked after 2 years when total weight loss (TWL) reached 35.1%. TWL was 28.6%, 28.4% and 26.7% after 10, 15 and 20 years. Mean body mass index decreased from 45.7 kg/m^2^ to 29.5, 32.5, 32.7 and 34.6 after 2, 10, 15 and 20 years respectively. Metabolic status remained improved after 20 years (reduced fasting glycemia, better lipid profile and lowered serum urates).

**Conclusions:**

RYGB is an efficient MBS procedure, associated with sustained weight loss and improvement of metabolic complications; a slight worsening occurs over time, partly attributed to aging and the chronic course of obesity and related metabolic disorders.

## Introduction

The prevalence of overweight and obesity is constantly increasing, leading to a rising number of patients submitted to metabolic/bariatric surgery (MBS) worldwide. MBS provides better weight loss than even the most potent recently introduced obesity management medications (OMM), the results of which remain largely unknown beyond 2–3 years. On the contrary, MBS has repeatedly been shown to provide significant and long-lasting weight loss with markedly enhanced quality of life, improvement and prevention of obesity-related complications, and increased life expectancy [1–4].

RYGB was first described in 1967. After some technical modifications, and especially since the advent of the laparoscopic approach, it rapidly gained in popularity and grew to represent the most performed bariatric procedure worldwide, until it was surpassed in numbers by sleeve gastrectomy about 10 years ago. Despite this long history, published long-term results are limited although several authors have reported on results up to 10 years after RYGB [3, 5–32]. Some studies, however, include only patients operated with open surgery or a mix of patients operated both openly or by laparoscopy and therefore do not represent current practice. In many studies, numbers of eligible patients and/or follow-up rates are limited after 10 years. Indeed, very few studies report 10-year results in a series of at least 100 patients with a follow-up rate ≥ 80% [3, 5, 6, 9, 18, 21, 32]. They typically show a mean 25–30% total weight loss (%TWL) after 10 years, with improved quality of life and good metabolic outcomes. After 15 or more years, results are even more scarce but show weight loss maintenance [3, 5, 6].

The aim of the present study is to report on 15–20-year results of laparoscopic RYGB performed as a first MBS procedure in a large cohort of patients operated over a 9-year period, with all patients being eligible for follow-up 15 years after surgery. The study focuses on weight loss, evolution of metabolic complications, and long-term procedure-related complications.

## Patients and Methods

Our bariatric database was searched for patients who underwent laparoscopic Roux-en-Y gastric bypass (RYGB) as a primary bariatric procedure between 1999 (first case) and December 2008 in one of our two reference bariatric centers lead by the same surgeon, so that all included patients have at least 15 years of follow-up. During the first years of this time period, we were still performing gastric banding, and RYGB initially was selected primarily for patients with BMI > 50. Due to satisfactory results with RYGB and because long-term complications requiring reoperation after gastric banding were increasingly common; however, RYGB soon became our most commonly performed precedure, so that all but 30 patients selected for MBS after 2001 underwent RYGB, meaning that the present cohort is mostly unselected. Patients were eligible for primary RYGB if they had a BMI > 40 or a BMI > 35 with obesity-related complications, had failed conservative therapy and had no contra-indication to surgery. Patients who underwent open RYGB and those with prior bariatric procedures were excluded. All patients were evaluated similarly by a multidisciplinary team before the indication for RYGB was confirmed, and all followed a 3-session preoperative course with information regarding the various bariatric procedures including their specific risks and consequences, dietary requirements after surgery and psychological aspects of obesity and massive weight loss.

The operative technique has been described elsewhere [33]. Briefly, it involved creation of a small 10–15-ml lesser curvature based gastric pouch with a linear stapler, division of the jejunum 30–50 cm below the angle of Treitz to form a short biliary limb and a 100–150-cm alimentary limb. The latter was brought up and anastomosed to the gastric pouch in a retro-colic and retro-gastric way using a 21-mm circular stapler, the anvil of which was introduced trans-orally. The jejuno-jejunostomy was performed with a linear stapler. Mesenteric windows were systematically closed, albeit the technique used for closure evolved over time [34].

Post-operative visits were scheduled after 1 month and every 3 months during the first post-operative year, every 6 months during the second year, and yearly thereafter. Patients not presenting for a scheduled appointment were systematically reminded by phone call and/or letter, and re-scheduled, unless their current address could not be found.

All initial data were retrieved from the bariatric chart. Follow-up data were mostly gathered during follow-up visits at the bariatric clinic, but also from electronic medical records if there were no follow-up data in the bariatric file. Patients who did not show up for follow-up visits were interviewed by phone and/or contacted by mail and invited to attend a formal face-to-face visit, unless they had moved to another country or their whereabouts could not be traced. For those who refused to attend, and with their permission, data was obtained from their general practitioner. Since weight loss was the primary endpoint, patients with no weight data for more than 12 months at the 10, 15 or 20-year terms, or without later weight data, were considered lost from follow-up, even if other information was available, such as lab tests or, for instance, emergency room visit record. Secondary endpoints were long-term procedure-related complications, mortality, and evolution of some metabolic blood markers (serum glycemia, lipid profile, uric acid). Factors potentially influencing weight loss were also analyzed.

Weight loss results are reported using %TWL and the Body mass index (BMI). To allow for easier comparison with other studies, we also report percentage of excess BMI loss (%EBMIL), with ideal weight considered as that corresponding to a body mass index of 25 kg/m^2^.

The creation of a bariatric database (COOL: COhort Obesité Lausanne) and its use for scientific research and clinical studies has been approved by the local ethics committee (CER-VD 304/15). All patients have given written permission for de-identified use of their data for scientific purposes. Data are reported in accordance with the STROBE guidelines [35].

Statistical analysis was done between groups using Student’s *t*-test for numerical variables and *Χ*^2^ or Fisher’s exact test for categorical variables. For factors found to be possibly associated with long-term weight loss on univariate analysis, relationship between variables was tested using logistic regression analysis to identify possible confounders. To assess the specific role of diabetes in this respect, a propensity-score matching model including all confounders was then used. Comparisons over time for continuous metabolic variables were done using analysis of variance (ANOVA) for repeated measures. A *p*-value < 0.05 was considered significant. All statistics were done using Stata SE 18.0 (StataCorp LLC, College Station, TX, USA).

## Results

A total of 944 patients, 717 females (76%) and 227 males with a mean age of 39.9 ± 10.6 years, underwent primary laparoscopic RYGB during the study period. The baseline characteristics of the patients are shown in Table 1.

**Table 1:** Patients’ details

Number of patients	944
Female (%)	717 (75.9)
Mean age (SD)	39.9 ± 10.6
Mean preoperative Weight (kg) (SD)	126.3 ± 21.6
Mean preoperative BMI (kg/m^2^) (SD)	45.7 ± 5.9
Patients with BMI ≥ 50 kg/m^2^	190 (20.1)
Diabetes (%)	190 (20.1)
Dyslipidemia (%)	675 (71.5)
Hypertension (%)	514 (54.4)
Obstructive sleep apnea syndrome (OSA) (%)	453 (48.0)

Overall early (0–30 days) morbidity was 11.5%, including a 2.8% major (Clavien-Dindo ≥ 3B) complication rate. One (0.1%) patient died during the immediate postoperative period as the consequence of a leak on the gastric remnant. All patients were eligible for follow-up after 10 and 15 years, and 340 were eligible after 20 years. Twenty-four patients died before the 10-year term, and 39 died before the 15-year term. Cardiovascular events and cancer (multiple types) were responsible for death in the majority of patients. Four patients committed suicide, and two died from drug overdose. Thirty-four of the 340 patients eligible for 20-year follow-up died before this limit. None of these late deaths was related to RYGB or a complication thereof. The overall mortality after 10, 15 and 20 years was therefore 2.9%, 4.1% and 10% respectively. The follow-up rates after these terms are 91.7, 74.5 and 52.3% respectively.

**Fig. 1:**
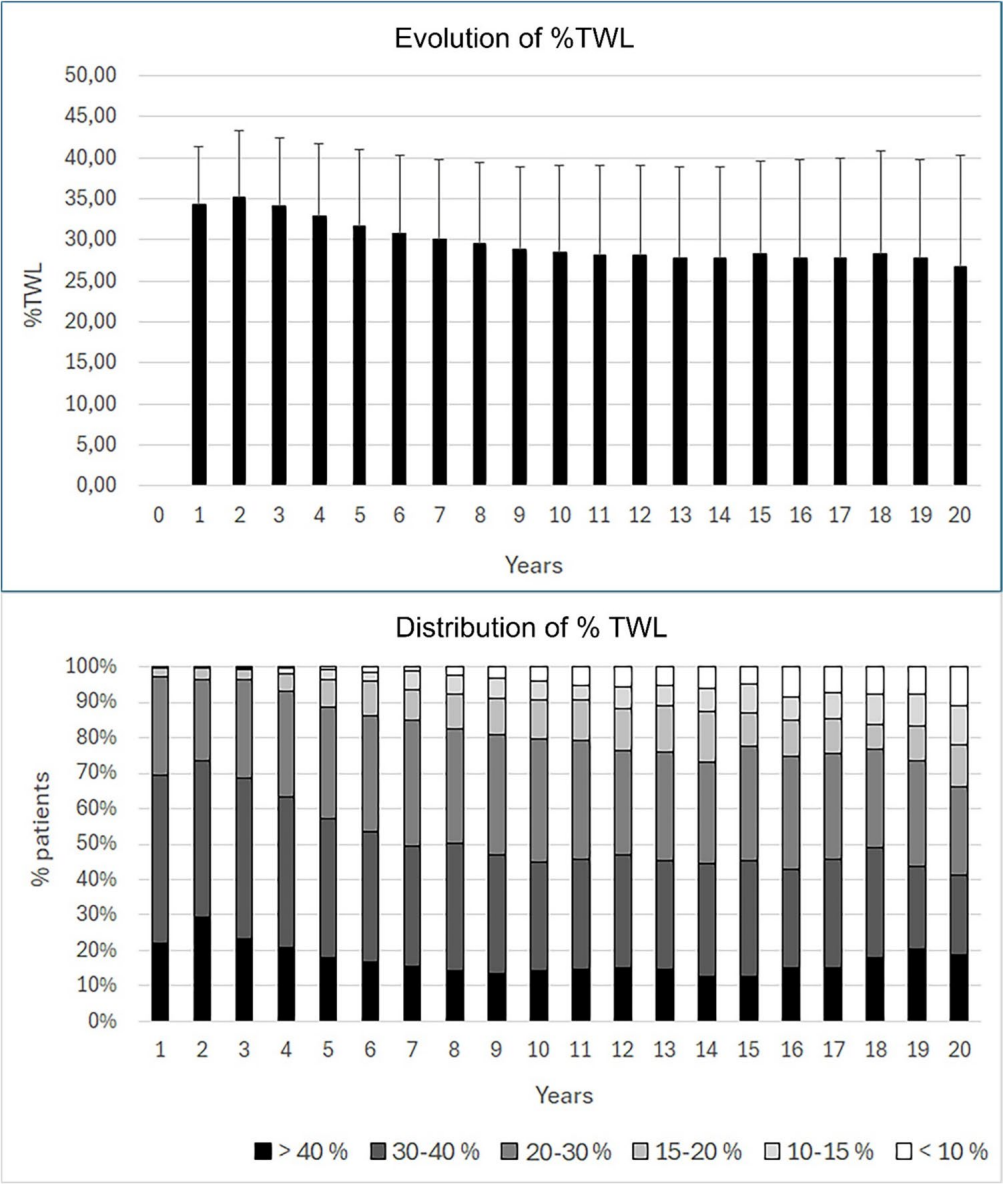
Evolution of %TWL and its distribution over time

The evolution of %TWL is depicted in Fig. 1. It was maximal after 2 years, at a mean of 35.1%, and very few patients (3.5%) had poor initial clinical response with TWL < 20%^34^. TWL decreased slowly afterwards, reaching 28.6% after 10 years, but remained relatively stable between 10 and 20 years, with 28.4% and 26.7% after 15 and 20 years respectively. After 10, 15 and 20 years, 79.4%, 77.8% and 66.4% of the patients had an optimal clinical response with TWL ≥ 20%. Overall, 75.7% of patients had some recurrent weight gain (RWG) over time. This remained limited and < 15% in 46% of cases but exceeded 30% in 35% of patients. After 15 years, 9.8% had TWL between 15 and 20%, and 7.8% had TWL between 10 and 15%. The 15-year TWL was < 10% in 4.5% of patients including 1.1% who had returned to their original weight or higher.

**Fig. 2:**
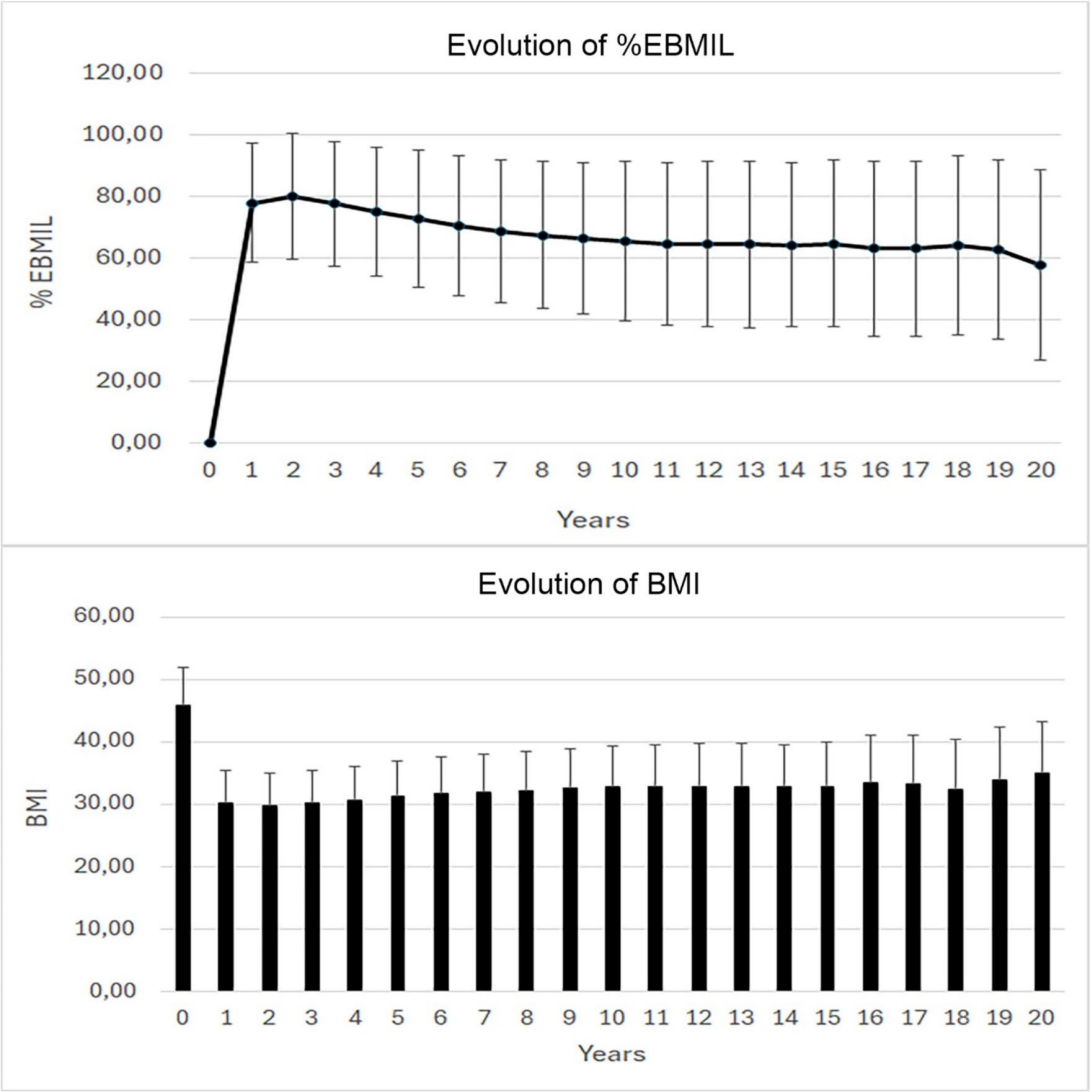
Evolution of %EBMIL and BMI over time

Figure 2 shows the evolution of BMI and %EBMIL. %EBMIL was maximum at 79.8 ± 20.4 after 2 years, 65.4 ± 25.8 after 10 years, 64.6 ± 27.2 after 15 years, and 57.6 ± 30.9 after 20 years, corresponding to a mean BMI of 29.5 ± 5, 32.5 ± 6.4, 32.7 ± 7 and 34.6 ± 8.2 after 2, 10, 15 and 20 years respectively. Patients with missing values after 15 years did not differ from patients with data in terms of BMI, sex, age and presence of obesity-related complications at baseline.

On univariate analysis, patients with diabetes lost significantly less weight during the first 6 post-operative years. On the contrary, they had better weight loss in the long term, with a significant difference between 15 and 17 years. Age < 55 was associated with significantly better early weight loss, but the difference disappeared afterwards, and patients older at surgery had even better weight loss after 19–20 years. Females lost significantly more weight than males up to 13 years after surgery and maintained a non-significant trend towards more weight loss later on. Other factors such as preoperative sleep apnea syndrome, hypertension, dyslipidemia, obstructive sleep apnea (OSA), osteoarticular pain, depression or initial BMI did not influence weight loss.

**Fig. 3:**
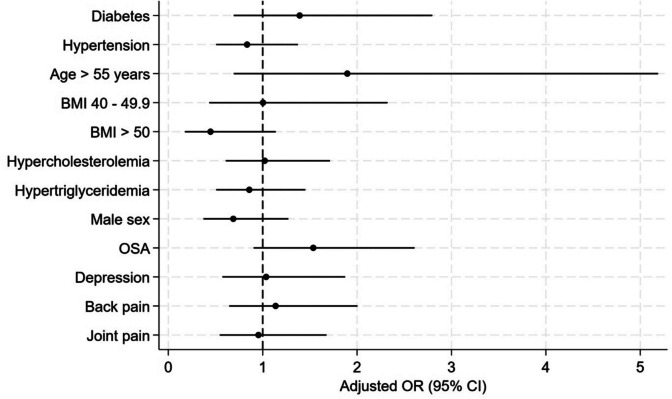
Forest-plot showing odds ratio (OR) for variables tested as possible predictors of long-term success (TWL ≥ 20%) in a logistic regression analysis. Reference group for BMI is < 40

After multivariate analysis in a logistic regression model including sex, age, diabetes status, BMI category, dyslipidemia, OSA, depression, osteoarticular pain, hypertension, no preoperatrive parameter was found to be associated with successful (≥ 20%) TWL after 15 years (Fig. 3). As diabetes is related to several other variables (age, sex, BMI, hypertension, sleep apnea), we used propensity scores to match patients with and without diabetes, and again concluded that diabetes is not an independent predictor of weight loss.

**Fig. 4:**
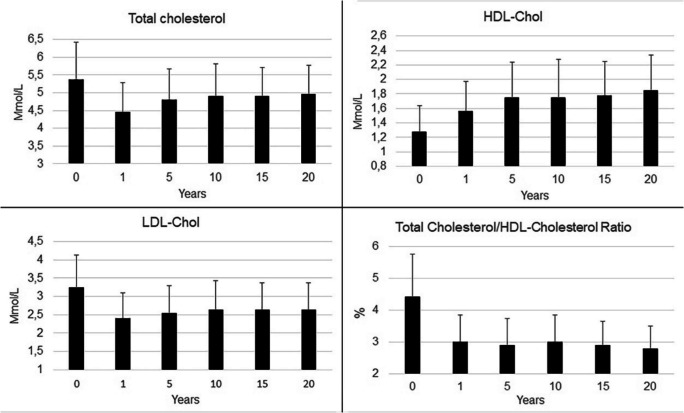
Evolution of total cholesterol, HDL-cholesterol, LDL-cholesterol and Total/HDL-cholesterol ratio over time. All values are significantly lower than before surgery at all time points with *p* < 0,0001 (ANOVA for repeated measures)

**Fig. 5:**
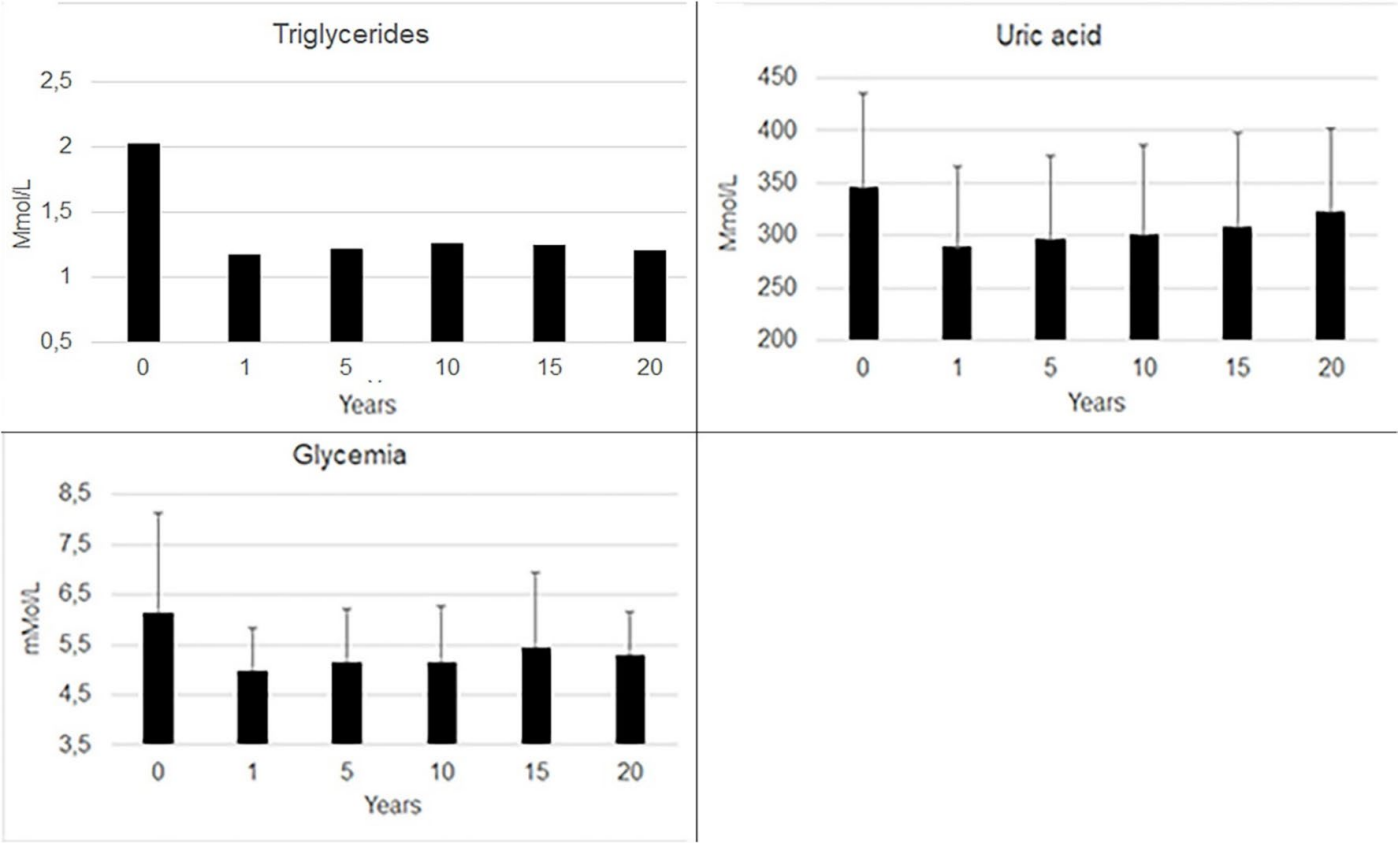
Evolution of triglycerides, uric acid and glycemia over time. All values are significantly lower than before surgery at all time points with *p* < 0,0001 (ANOVA for repeated measures)

The lipid profile improved rapidly during the early years after RYGB and remained significantly improved up to 20 years post-surgery. In patients with RWG ≥ 30% after 15 years, however, and while remaining significantly lower than before surgery, 15-year lipid values were higher than in patients with RWG < 30%. Figure 4 shows the evolution of total cholesterol, HDL- and LDL-cholesterol and total/HDL cholesterol ratio over time, while the evolution of triglycerides is depicted in Fig. 5. Uric acid and glycemia also improved over the entire follow-up period, as shown in Fig. 5. Glycemia was not impacted by RWG after 15 years.

Long-term complications developed in 178 patients beyond the first postoperative month (Table 2).

**Table 2:** Long-term complications (> 30 days post-surgery)

Type of complication	Number	%
Stricture at the gastrojejunostomy	38	4.0%
Internal hernia	81	8.5%
Marginal ulcer	14	1.5%
Intestinal obstruction/sub-occlusion	24	2.5%
Incisional hernia	9	0.9%
Recurrent abdominal pain	13	1.3%
Symptomatic cholelithiasis	5	0.5%
Candy cane syndrome	7	0.7%
Common bile duct stones	1	0,1%
Hiatus hernia	5	0.5%
Protein malnutrition	1	0.1%
Myopathy caused by nutritional issues	1	0.1%
Small intestinal bacterial overgrowth	1	0.1%
Intussusception	3	0.3%
Total patients with late complication(s)	178	18.5%

**Fig. 6:**
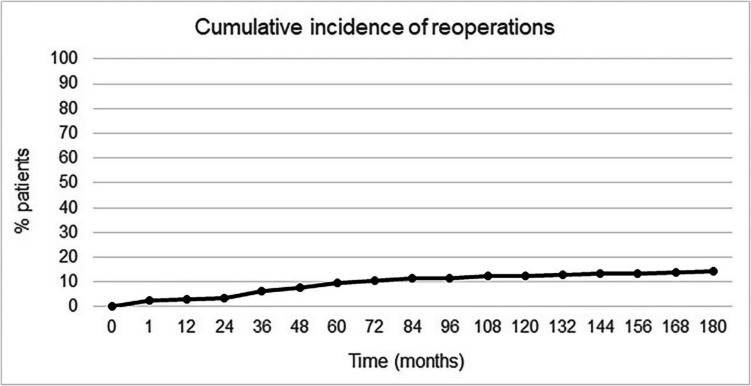
Cumulative incidence of reoperations over time

Stricture at the gastrojejunostomy developed in 38 patients between 3 and 6 postoperative weeks and was treated successfully with endoscopic dilatation in all cases. Internal hernia (IH) affected 81 (8.5%) patients and was the most common late complication, leading to partial or total intestinal obstruction in 28 (2.9%) cases. All these IH were treated operatively. Another 35 (3.7%) patients required laparoscopy either for recurrent intermittent abdominal pain or for bowel occlusion/sub-occlusion due to adhesions or bands or a bezoar in one case. A total of 134 (14.2%) patients were reoperated during follow-up (Table 3). Figure 6 shows the cumulative incidence of reoperations during the first 15 years.

**Table 3:** Long-term endoscopic and surgical reinterventions (ERCP: Endoscopic retrograde cholangiopancreatography)

Type of re-intervention	Number	%
Endoscopic dilatation for stricture	38	4%
Incisional hernia repair	10	1%
Correction of internal hernia	81	8.5%
Laparoscopy/tomy for pain or occlusion	35	3.7%
Cholecystectomy	5	0.5%
Resection of candy cane	7	0.7%
Hiatus hernia repair	5	0.5%
Reduction of intussusception	2	0.2%
Transgastric ERCP with stone removal	1	0.1%
Gastrojejunostomy revision	1	0.1%
Closure of gastric remnant perforated ulcer	1	0.1%
Partial resection of biliary limb	1	0.1%
Total patients with surgical reintervention	134	14.2%

## Discussion

To the best of our knowledge, the present study so far is the largest reporting on results 15–20 years after primary laparoscopic RYGB in patients with severe obesity. As shown before, the nadir weight is achieved around 2 years after surgery, followed by RWG mostly between 2 and 10 years, and stabilization thereafter. Reports on 15-year results after RYGB are scarce and include only limited number of patients. Angrisani et al. reported a 26.8% mean TWL in a series of 105 patients [5]. In the Swedish Obese Subjects study, TWL was 27.2% in 37 patients [2]. McClelland et al. reported a 28% TWL in 92 patients [6], and Jawhar et al. demonstrated 25.6% in 85 patients [7]. Numbers are even smaller after 20 years, with a mean %TWL of 26% in the SOS study (13 patients) and 24.3% in the study by McClelland (12 patients). The baseline characteristics of the patients included in these studies are slightly different from those from the present series, but the surgical technique, when described, is similar. Overall, however, the differences in terms of baseline BMI and age are limited and should not prevent a fair comparison. The present study, with a much larger group of patients and higher long-term follow-up rates, confirms that RYGB provides significant and durable weight loss up to 20 years after surgery. Most patients maintain a long-term TWL > 20%, although the percentage decreases over time. TWL < 10% after 15 years affects few patients. We are convinced that regular follow-up by both surgeons and other members of the multidisciplinary team contributes to these satisfying long-term results.

In the SOS study, Sjôstrôm et al. have shown that conventional medical therapy does not result in long-term weight loss [2]. Newer modern obesity management medications (OMMs) have shown promising short-term weight loss, but little is known about mid-term results and nothing about long-term effects. OMMs are not tolerated by everyone, are currently expensive, are often not reimbursed by the healthcare system, or only for a limited period of time as in our country. In any case, the results of the present study after RYGB are superior even to currently available OMMs. Currently, we consider OMMs more as complementary to RYGB than as a single treatment for patients with obesity class 2 and more whose response to surgery is suboptimal.

Long-term weight loss results after RYGB compare well with those reported after sleeve gastrectomy (SG), currently the most popular bariatric procedure worldwide, although not in our country. A recent review of results 10 or more years after SG showed an overall weighted TWL of 24.4% (17–36.7). It also showed a weighted 32.3% (21.4–58.4) prevalence of persisting or de novo long-term gastro-esophageal reflux disease (GERD), and the weighted long-term reoperation rate was 19.2% [36]. Although not totally absent and not reported in this paper, GERD is unusual after RYGB. The results from the present series are superior to SG in terms of weight loss and reoperation rate. On the other hand, hypoabsorptive procedures such as biliopancreatic diversion (BPD) with or without duodenal switch (BPD-DS) lead to an average 10% better long-term and more durable TWL than RYGB, but at the price of more frequent and more severe long-term nutritional complications [37–40].

During the first few years after surgery, male sex, increased age and the presence of diabetes negatively influence weight loss. The influence of sex of weight loss varies in the literature. Many authors report better weight loss in females [6, 13, 41], as in the present study, but others show no difference between sexes [7, 14, 15]. In a study involving only patients with class 4 obesity, our group reported better weight loss in male patients [12]. Regarding age at baseline, except for few authors claiming better weight loss early after surgery in younger patients [11, 42], most studies fail to report any difference between older and younger patients in the long term [11, 15–17]. Our findings are consistent with those of Monaco-Ferreira et al., who also showed that younger patients regain more weight than older ones in the long term [17]. In patients with preoperative diabetes, reduced weight loss in the short- and mid-term after RYGB, as in the present series, has often been reported [43–46], but findings are not uniform, and other studies show no influence of diabetes on weight loss [47]. In the present study, diabetes was found to be associated with better long-term weight loss on univariate analysis. This was a new finding, for which we could not find any explanation, and no similar trend could be found in the literature. Logistic regression analysis as well as propensity score matching taking into account possible confounders (age, BMI hypertension, sleep apnea, sex), however, failed to confirm an independent role for diabetes in long-term weight loss.

An improved lipid profile including reduced triglycerides, total and LDL cholesterol levels and increased HDL cholesterol levels has been documented by several groups during the early years after RYGB, and has been confirmed in a meta-analysis in 2016 [8–10, 48–51]. These changes are similar across obesity classes or age at baseline and persist despite some RWG [11, 12, 48]. The present study confirms these findings and shows that the positive early changes persist in the very long term. Total cholesterol achieves its nadir one year after surgery, re-increases slowly thereafter but remains constantly inferior to preoperative levels. The LDL-cholesterol fraction follows the same pattern whereas triglycerides level drops permanently. Interestingly, HDL-cholesterol increases constantly after surgery, even between 2 and 10 years after surgery, when most RWG occurs, so that the Total/HDL cholesterol fraction remains much better and unchanged during the entire follow-up duration. These changes in lipid values are even more remarkable considering that our population is aging by 15–20 years during the study period, the mean age increasing from 40 to 60 years. Lipid values are known to be normally increasing during this time interval [52], so that part of the evolution seen after nadir in the present series is probably attributable to aging. These improvements have been associated with reduced cardiovascular risk [49] and a decreased use of lipid-lowering medications [53].

In the present study, we did not evaluate the evolution of HbA_1_c since values at baseline are missing for most patients. Information regarding use of antidiabetic medications after 15–20 years is also missing, thus a formal analysis of complete or partial diabetes remission rate is impossible. Many authors have reported better control of diabetes with reduced HbA_1_c up to 12 years after surgery, and a significant remission rate, although the latter decreases with time [54]. As for lipids, aging is associated with an increasing prevalence of diabetes and hyperglycemia [55], so that within a period of 15–20 years, one would normally expect global deterioration. While a detailed analysis is not possible, the improved fasting glycemia that persists for 20 years in this study reflects better overall control of glycemia in the entire cohort, and very likely persistent remission of diabetes in a subset of patients who had T2D at baseline.

A recent review showed that, after an initial increase during the early postoperative phase, hyperuricemia decreases after BMS in association with a reduced number of gout flares [56]. As long-term uric acid levels remained reduced, the present study suggests that RYGB also has a long-lasting protective effect against gout.

The overall mortality observed in the present study needs to be interpreted with caution beyond the first 10 years after surgery, because of incomplete follow-up. The 4.1% 15-year and 10% 20-year mortality reported herein, however, compare well with figures from the literature, notably those reported recently in a large meta-analysis where mortality in surgical patients was 5% and 8.8% after 15 and 20 years respectively, significantly lower than the 12.4% and 20% observed in matched non-operated patients [1].

All patients from this cohort required various micronutrient supplementations during follow-up in addition to a basic multivitamin supplement. A significant number of patients also developed other long-term complications, some requiring one or more reoperations. Many complications observed during follow-up were in part related to our early experience performing RYGB and its associated learning curve. The 3 most common long-term complications were internal hernia (IH) ± small bowel obstruction (SBO), stricture at the gastro-jejunostomy (GJS) and SBO unrelated to IH. Since 2006, a modification of the technique used to perform the GJS has almost completely suppressed strictures at this level despite the anastomosis still being performed using a 21-mm circular stapler [57]. More recently, and not yet reflected in the present series because it was introduced only in 2008, improved and meticulous technique to close the mesenteric defects after completion of RYGB, using running non-absorbable sutures and including a purse-string suture on the posterior aspect of the jejunojejunostomy defect, has led to a markedly reduced rate of IH (1.05%) and IH-related bowel obstructions [34]^32^. IH, however, cannot be completely prevented, and neither can SBO due to adhesions, so that some reoperations for these problems will remain necessary. Marginal ulcers were uncommon in this series, probably due to the very small gastric pouch and avoidance of NSAIDS. The risk to develop a candy-cane below the GSJ can be minimized by leaving a very short afferent loop when dividing the jejunum after completion of the GJS. Very few patients required cholecystectomy during follow-up, but almost routine concomitant cholecystectomy in the presence or not of gallbladder stones was the rule in the present series.

Despite these good overall results, a small percentage (1.3%) of patients never achieve the 20% TWL limit, and a minority of patients (3.5%) have a poor initial clinical response with TWL < 20% after 2 years [58]. Although about one-fourth of the latter have better TWL after 10 and 15 years than initially, most will not improve in the long term. This may be partly related to the short biliary limb used in this series. Currently, hoping that it will contribute to better results, we adjust the length of the biliary limb according to preoperative BMI and diabetes status, and use a longer (100–150 cm) biliary limb in patients with initial BMI > 50 and/or diabetes making sure, however, that the common limb length remains at least 300 cm with a 100-cm alimentary limb. Nowadays, patients with suboptimal clinical outcomne are candidates for OMMs. They could also be submitted to reoperation with elongation of the biliopancreatic limb, a technique that can provide a further 24% BWL after 3 years [59]. These two same options are available for patients with excessive RWG, especially with recurrence of obesity-related complications. Other techniques such as conversion to biliopancreatic diversion with duodenal switch (BPD-DS) or sleeve gastrectomy with single-anastomosis duodeno-ileostomy (SADI-S) can also be considered in selected patients but are associated with higher perioperative morbidity and long-term nutritional risks.

Strengths of this study are the large number of patients and the good follow-up rates, at least up to 15 years. The results presented herein should therefore be representative of the entire series, especially since the minority of patients with missing data regarding weight loss after 15 years did not differ significantly from patients with available data regarding values at baseline for age, sex, BMI, and prevalence of several obesity-related complications (diabetes, hypertension, SAS, dyslipidemia, depression, joint pain).

The present study also has limitations. Some important data such as medications before RYGB and at follow-up, as well as HbA1_c_ at baseline, are missing. Serum lipids and glycemia are therefore used as surrogates to evaluate obesity-related complications, which means that even if normal, they cannot necessarily be considered as reflecting remission. Despite the fact that a subset of patients were treated for diabetes or with lipid-lowering medication before surgery, and some still receive or have been started with medication at a later stage, long-term improved glycemia and lipid profile reflect better control of these conditions, which should contribute to reduced cardiovascular risk profile and mortality. Another limitation is the absence of a comparison group. The present results, however, match those reported in large surgical cohorts from comparative and non-comparative studies in the literature.

## Conclusions

The present study is the first to report on a large group of patients submitted to RYGB with 15–20-year results and confirms results from previous smaller studies. RYGB is associated with a mean TWL between 25 and 30% up to 20 years after surgery, and improved metabolic values reflecting better control of obesity-related metabolic complications. These results add to the body of evidence regarding the long-term effectiveness of RYGB for the treatment of patients with severe obesity. Ideally, confirmation should be obtained in a randomized controlled trial comparing RYGB with best medical treatment and/or other surgical procedures, ideally multicentric, and very long-term follow-up in a large group of patients.

## Data Availability

Aggregated data is provided within the manuscript. Detailed data in the form of Excel file can be provided upon request.
